# Psychodynamic psychotherapy for schizophrenia spectrum disoders: a case presentation and systematic review

**DOI:** 10.1192/j.eurpsy.2023.2178

**Published:** 2023-07-19

**Authors:** J. N. Kjær, B. Rosenbaum

**Affiliations:** 1 Psychosis Research Unit, Aarhus University Hospital - Psychiatry, Aarhus N; 2 Institute of Psychology, University of Copenhagen, Copenhagen, Denmark

## Abstract

**Introduction:**

Psychodynamic psychotherapy emphasizes the unique history, subjectivity, and psychological complexity of each individual. The core principles in the psychodynamic treatment of schizophrenia spectrum disorders (SSD) includes a stable (yet flexible) frame; attention to countertransference; and clarification of experiences, emotions, and relationships including giving psychotic symptoms context in internal and external object relationships.

**Objectives:**

This study has two aims. First, to present the progress of a patient with chronic schizophrenia treated with psychodynamic psychotherapy. Second, to provide a systematic review of comparative trials that have included psychodynamic psychotherapy as treatment for SSD.

**Methods:**

The case presentation includes information from the therapist’s notes, video footage, and the electronic health record. The systematic review will be conducted in November and December, 2022, and in accordance with the Preferred Reporting Items for Systematic Reviews and Meta-Analyses 2020 guidelines. The databases of MEDLINE, EMBASE, and PsycInfo will be searched for literature.

**Results:**

L, a 25-year-old woman, was diagnosed with paranoid schizophrenia five years prior to starting psychodynamic psychotherapy at an outpatient unit for SSD. L grew up with a close relationship with her mother, father, and sister. She was bullied in school and clearly remembered being told by her classmates that she was “just L”. When therapy began L had been living with her boyfriend for six months. Voice hallucinations were one of the most interfering symptoms. The most present voice, M, was both her best friend and worst enemy. During psychotic breakdowns M could take control of L’s body. L was incapable of making her own decisions. Small and big decisions were consulted with a family member or the hallucinatory voices. L attended 33 psychotherapy sessions from October, 2020 to November, 2021. In the first six months, sessions were weekly and afterwards biweekly due to L feeling significantly better and she wanted to have more time to study. L benefitted from the structure and clarifying questions from the therapist. Most notably, she broke up with her boyfriend. She started dating and found a new boyfriend. At this point the voice hallucinations and psychotic breakdowns were reduced considerably. In the termination phase the themes were feeling insecure, relationships, and how having been bullied affected her as an adult.

Results from the systematic review are not available at the time of submission.

**Conclusions:**

In the present case, psychodynamic psychotherapy was an effective treatment of psychotic symptoms as well as childhood trauma and interpersonal conflicts for an individual with paranoid schizophrenia. It speaks for a broad application of psychodynamic psychotherapy in the treatment of SSD as the therapy both assesses and treats psychotic and non-psychotic symptoms.

**Disclosure of Interest:**

None Declared

